# Dual-targeted photothermal agents for enhanced cancer therapy

**DOI:** 10.1039/d0sc03173a

**Published:** 2020-07-17

**Authors:** Kaiye Wang, Yanan Xiang, Wei Pan, Hongyu Wang, Na Li, Bo Tang

**Affiliations:** College of Chemistry, Chemical Engineering and Materials Science, Key Laboratory of Molecular and Nano Probes, Ministry of Education, Collaborative Innovation Centre of Functionalized Probes for Chemical Imaging in Universities of Shandong, Institute of Molecular and Nano Science, Shandong Normal University Jinan 250014 P. R. China lina@sdnu.edu.cn tangb@sdnu.edu.cn

## Abstract

Photothermal therapy, in which light is converted into heat and triggers local hyperthermia to ablate tumors, presents an inherently specific and noninvasive treatment for tumor tissues. In this area, the development of efficient photothermal agents (PTAs) has always been a central topic. Although many efforts have been made on the investigation of novel molecular architectures and photothermal materials over the past decades, PTAs can cause severe damage to normal tissues because of the poor tumor aggregate ability and high irradiation density. Recently, dual-targeted photothermal agents (DTPTAs) provide an attractive strategy to overcome these problems and enhance cancer therapy. DTPTAs are functionalized with two classes of targeting units, including tumor environment targeting sites, tumor targeting sites and organelle targeting sites. In this perspective, typical targeted ligands and representative examples of photothermal therapeutic agents with dual-targeted properties are systematically summarized and recent advances using DTPTAs in tumor therapy are highlighted.

## Abstract 2

In this perspective, the design, applications and potential directions of photothermal therapeutic agents with dual-targeted properties are systematically described.

## Graphical abstract

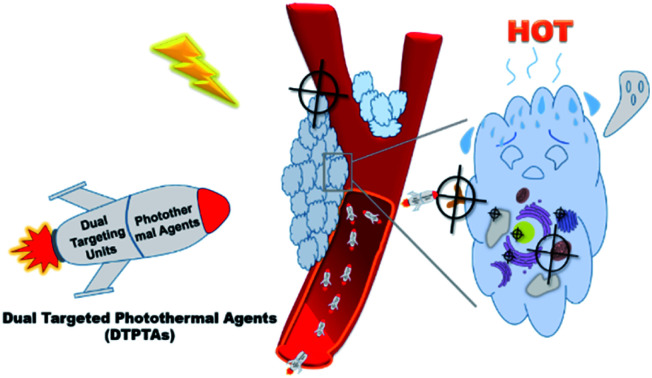

## 1. Introduction

Cancer, as one of the most intractable diseases, has spoiled millions of lives due to its high morbidity and mortality.^1,2^ Over the past decades, great efforts have been focused on the development of efficient therapeutics for cancer treatment, such as surgery,^3^ radiotherapy,^4,5^ chemotherapy,^6,7^ photodynamic therapy (PDT),^8–10^ photothermal therapy (PTT)^11–13^ and immunotherapy.^14–16^ In particular, PTT has attracted extensive attention due to its inherent advantages of minimal invasion and external light control. According to the mechanism of PTT for cancer therapy, it achieves the goal of killing cancer cells *via* converting light energy to heat in order to trigger local hyperthermia in tumor, so PTT does not suffer from the limitations in hypoxic environments compared to PDT. Up to now, PTT as an important and promising treatment option has been a focus of attention to develop potential photothermal agents (PTAs) over the past decades. A broad range of novel inorganic and organic materials have been designed as PTAs to ablate tumor tissues. Although great advances have been made in the development of PTAs, it has been found that hyperpyrexia can seriously damage surrounding normal tissues because of poor tumor aggregation and high intensity irradiation. Hence, the development of efficacious PTAs with excellent tumor inhibition and negligible side effect presents a challenging task for cancer therapy.

**Scheme 1:**
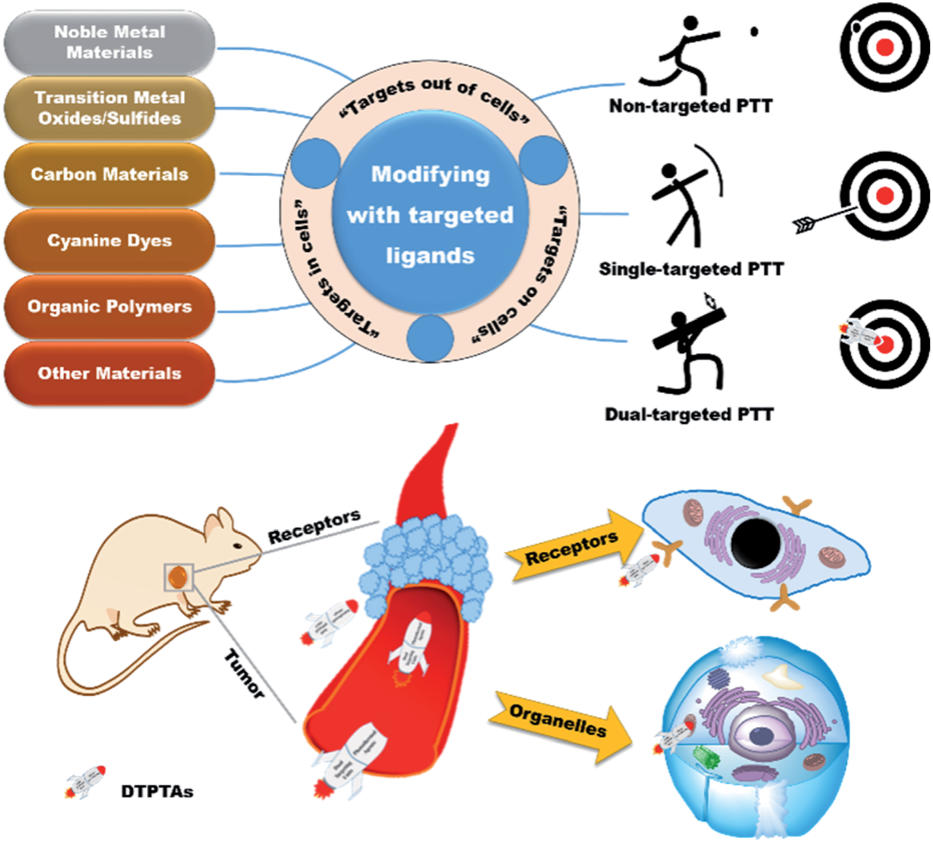
Overview of dual-targeted PTT.

The rapid growth and proliferation of tumors result in abnormal demand and metabolism, which is beneficial for distinguishing normal tissues from diseased tissues. Therefore, targeted therapeutic agents exhibit enormously potential applications in PTT and have received extensive attention.^17,18^ Unfortunately, although single-targeted PTAs have made great achievements in cancer therapy, it is nonnegligible that off-targeted activities occur occasionally due to the dynamics and saturability of receptors on cancer cells.^19,20^ For further access to the tumor tissues, dual-targeted photothermal agents (DTPTAs) have been developed to ablate solid tumors, with slightly adverse effects (Scheme 1).^21^ Modified with two different targeted groups, DTPTAs show highly precise locating capability. Besides this, DTPTAs can trigger cell necrosis at low temperature and cause negligible damage to surrounding tissues when one of the targeted ligands tends to aggregate in the organelle, where heat-sensitive proteins and DNA are enriched.

In this perspective, typical targets are classified from three aspects based on the position of recognition sites, namely “extracellular targets”, “intracellular targets” and “subcellular targets” (Table 1). Furthermore, the applications of dual-targeted photothermal materials are carefully introduced. DTPTAs are described on the basis of different materials, including noble metal materials, transition metal oxide/sulfide materials, carbon materials, cyanine dyes, and organic polymers, as well as other materials. Photothermal conversion capability and tumor inhibition are also described in detail.

**Table 1:** Specific sites or stimulation and targeting moieties

Typical targets	Specific sites or stimulation	Targeted moieties
Extracellular targets	Hypoxia	Modified anaerobions^22–24^
		Anti-CCL-28^a^^,^^25^
		Anti-LDLR^b^^,^^26^
		Tirapazamine^27^
	Acidity	Hydrolysis^28^
		Protonation^29^
		Hydrophilic transformation^30^
		pHLIP^31,32^
	VEGF	Anti-VEGFmAb^33^
		Bevacizumab^34^
	VEGFR	VEGFR-2 antibody^35^
	MMP	GPLGVRGC^36^
		PG-6 (ref. 37)
	Magnetic field	Fe_3_O_4_ (ref. 38)
Intracellular targets	Integrin α_v_β_3_	RGD^39–42^
		Integrin α_v_β_3_ mAb^43,44^
	EGFR	CET^45,46^
		Gefitinib^47,48^
		Erlotinib^49,50^
	FR	FA^51–53^
		MTX^54^
	BR	Biotin^55,56^
	TfR	Tf^57,58^
	CD44	HA^59,60^
Subcellular targets	Nucleus	NLS^61^
		TAT^62^
		AS1411 DNA aptamer^63^
	Mitochondria	TPP^64,65^
		Cyanine cation^66^
		MLS^67^
	Lysosomes	Morpholine^68^
	ER	Sulfamides^69^
		Pardaxin (FAL) peptides^70^

a Anti-CCL-28: a ligand that targets hypoxia of the overexpressed CCL-28 chemokine.

b Anti-LDLR: a ligand that targets hypoxia of the marked low-density lipoprotein receptor.

### 1.1. “Extracellular targets”

“Extracellular targets” refers to recognition sites present in the tumor microenvironment (TME). Because of the vigorous metabolism and rapid growth of the solid tumor, extreme environments, including hypoxic environments, acidic environments, angiogenesis and overexpression of enzymes, are generated in the surrounding tumor tissues. By taking advantages of these differences between normal tissues and cancers, extensive investigations have been made for the specific treatment of tumors.^71,72^

#### Hypoxic environment

Although solid tumors contain intricate veins, oxygen and glucose are still in short supply due to their expanding tumor tissues and growth size.^73,74^ As a result, a hypoxic environment is generated around the tumor tissues. To target this hypoxic environment, genetically modified anaerobions have become important carriers based on anaerobic orientation.^22–24^ Additionally, typical ligands such as anti-CCL-28,^25^ anti-LDLR^26^ and hypoxia-specific drug tirapazamine^27^ have also been applied to target hypoxia.

#### Acidic environment

The hypoxic environment of the tumor prevents adequate oxidation of glucose, which promotes tumor cells to derive energy not only from oxidative phosphorylation but also from glycolysis. During the process of glycolysis, a large amount of lactic acid is produced and excreted into the TME, resulting in an acidic environment. Various changes are prone to occur in an acidic environment, such as hydrolysis reactions;^28^ protonation^29^ and hydrophilic transformation.^30^ In addition, the pH low insertion peptide (pHLIP) can transform into an regular α-helix in acid environment to insert into membrane, so it can be used for targeting tumor cells.^31,32^

#### Angiogenesis

To relieve anoxia, vascular endothelial growth factor (VEGF) is released from cancer cells into the TME to promote angiogenesis. VEGF can specifically combine with VEGF receptor (VEGFR) on the surface of surrounding endothelial cells, so both VEGF and VEGFR are potential targets for cancer therapy. Anti-VEGF monoclonal antibodies (anti-VEGFmAb), which behave amiably with VEGF were widely used for targeted PTT.^33^ The antibody bevacizumab also shows the ability to accurately identify VEGF.^34^ In addition, modified with the VEGFR-2 antibody, PTAs have also been developed to promote powerful TME aggregation and photothermal effect.^35^

#### Matrix metalloproteinase (MMP)

The dense extracellular matrix of the TME is mainly composed of collagen for slowing tumor invasion, migration and angiogenesis. To deal with this issue, carcinoma cells stimulate stromal fibroblasts and endothelial cells to secrete MMPs to degrade extracellular matrix. Multiple MMP subspecies including MMP-2, MMP-9, MMP-13 and MMP-14, can cut specific GPLGVRGC sequences, which have been widely applied for cancer stimulation therapy.^36^ Besides this, the PG-6 peptide (PLGALG)^37^ is another MMP discriminating peptide that can be identified and cut by the MMP-2 and MMP-9 subspecies.

#### Magnetic field

Magnetic targeting is a method for artificially controlling tumor aggregation based on an external magnetic field. Magnetic materials, such as iron oxide nanoparticles (IONPs)^38^ have been widely used for anti-cancer treatment due to their properties, such as non-toxicity, small size, and easy functionalization. In addition, magnetic materials demonstrate strong imaging capabilities in magnetic resonance imaging (MRI), which is beneficial to guiding tumor treatment. The released iron ions can also catalyze the Fenton reaction to produce reactive oxygen species (ROS) for chemodynamic therapy. Moreover, the good biocompatibility, biodegradability, pharmacokinetics and bimodality of magnetic particles exhibit great potential applications in clinical trials.

### 1.2. “Intracellular targets”

“Intracellular targets” means that receptors exist on the surface of the tumor cytomembrane. Because cells are relatively separate spaces, substance ingestion requires the assistance of receptors that are embedded in the cell membrane, while specific receptors are produced to transfer different substances, including ions, small molecules and biomacromolecules. Tumor cells have abnormal metabolism, so many receptors are over-expressed. Many efforts have been focused on the development of specific drug-delivery systems based on these over-expressed receptors over the past decades.

#### Integrin α_v_β_3_

Integrins are crucial heterodimeric receptors for cell adhesion and intracellular signal transduction, then mediate tumor growth and invasion. Integrin α_v_β_3_ as one of the integrin subspecies is closely associated with angiogenesis, wherein integrin α_v_β_3_ is overexpressed in tumor tissues. A cell binding peptide RGD (arginine–glycine–aspartate) has been known to be able to identify integrin α_v_β_3_. Due to its advantages of being modifiable by simple chemical synthesis, it has been recognized as the most commonly used targeting ligand over the past few decades. In addition, the monoclonal antibody against α_v_β_3_ (integrin α_v_β_3_ mAb) is also an important target to achieve the goal of targeting tumor cells. For this reason, various PTAs conjugated with RGD^39–42^ or integrin α_v_β_3_ mAb^43,44^ have been applied for tumor targeted PTT.

#### Epidermal growth factor receptor (EGFR)

EGFR as a kind of receptor is overexpressed on the surface of multiple tumor cells. Moreover, it can specifically combine with epidermal growth factor, which is a cell growth factor with high physiological activity. After binding to the ligand, EGFR can inhibit apoptosis and trigger a series of enzymatic signalling to enhance cell growth, invasion, and also migration. Moreover, EGFR can specifically bind to monoclonal antibodies EGFRmAb (Cetuximab, CET)^45,46^ to achieve internalization. In addition, as a tyrosine kinase receptor, EGFR can be inhibited by corresponding small molecule inhibitors, such as gefitinib^47,48^ and erlotinib.^49,50^ Both monoclonal antibodies and inhibitors inhibit EGFR-mediated downstream signalling when providing targeting capabilities.

#### Folate receptor (FR) and biotin receptor (BR)

Due to the rapid proliferation of malignant cells, vitamins are highly demanded to promote cell growth. For example, folate (FA) is mainly involved in cell growth and the formation of DNA, which enters carcinoma cells mediated *via* the FR. In order to bind and internalize FA, FRs are over-expressed on the surface of tumor membranes. Another typical vitamin in high demand is biotin (also called vitamin H), which must be ingested in the daily diet of humans. Both FA^51–53^ and biotin^55,56^ as natural small molecules have shown advantages of being low cost, biologically stable and easy to modify, so they have been widely used as targeted groups. In addition, the chemotherapeutic agent methotrexate (MTX) also shows an affinity to FR because of it having a similar structure to that of FA.^54^

#### Transferrin receptor (TfR)

A large amount of iron ions are needed to be transferred into cancer cells in order to synthesize heme for carrying oxygen. In this process, TfR as a kind of glycoprotein embedded in membrane has shown the significant responsibility of binding transferrin (Tf) and endocytosis of Tf, which accelerates transport and internalization of iron ions *in vivo*. Meanwhile, tumor cells express TfR in high levels, which is beneficial to cancer-targeted therapy. Through the camouflage of Tf, plenty of nano-photothermal therapeutic agents have been used for targeting tumor cells.^57,58^

#### Cluster of differentiation-44 (CD44)

In the cluster of differentiation family, the CD44 receptor is a kind of transmembrane glycoprotein for extracellular matrix adhesion and signal transduction, as well as stem cell migration. Besides this, many studies have shown that CD44 can serve as a marker for cancer stem cells, which are closely associated with tumor recurrence, migration, and drug resistance due to their outstanding self-regulation. Hyaluronic acid (HA), as an endogenous polysaccharide, binds well to CD44 and then is mediated to internalize into cells. For these reasons, HA modified therapeutic agents^59,60^ have been widely developed and applied in tumor-targeted therapy and are considered to be an effective method to inhibit tumor metastasis.

### 1.3. “Subcellular targets”

“Subcellular targets” means that PTAs are conjugated with organelle-targeted ligands, so the photothermal materials can efficiently induce apoptosis of organelles after endocytosis. Because of heat-sensitive proteins and genetic material in organelles, organelles are easily destroyed and cause cell death at low-dose irradiation, thereby reducing damage to surrounding normal tissues. Hence, organelle-targeted imaging and therapy have attracted wide attention in exploring technologies for tumor inhibition and life regulation.^64,75,76^

#### Nucleus

The nucleus as the brain of cell contains most of the genetic material to control the replication and expression of genes, and takes part in multiple processes such as cell growth, division and apoptosis. Tumorigenesis is based on genetic mutations, which make the nucleus become an ideal target for tumor suppression. However, such an important organelle is protected by a two-layer-membrane, and the exchanges of materials mainly depend on the strict control of nuclear pores complex. Nuclear localization sequence (NLS) peptides^61^ and TAT peptides^62^ have been demonstrated to have excellent capability of targeting the nucleus, which have been exploited to overcome the difficulty of poor nuclear localization. Aptamers based on artificially screened oligonucleotide or polypeptide sequences have the advantage of highly specific targeting properties. The AS1411 DNA aptamer^63^ exhibits excellent nuclear recognition and has been used for nuclear localization therapy.

#### Mitochondria

Mitochondria, where high-energy ATP is generated *via* complex reactions, are recognized as the energy-supplying organelles. Although glycolysis provides a large amount of energy to the cancer cells, mitochondria is involved in a variety of biological processes such as the maintenance of oxidative phosphorylation. The destruction of mitochondria will lead to the apoptosis of cells, so it has been regarded as an important target for cancer therapy. Extensive investigations illustrated that triphenylphosphonium (TPP) exhibited good mitochondrial aggregation by taking advantage of hydrophobicity and positive charge, thus TPP has been widely explored for guiding targeted therapy.^64,65^ Meanwhile, cyanine cation^66^ and mitochondria localization (MLS) peptides^67^ can also target mitochondria, which have been applied to transport PTAs to mitochondria for cancer therapy.

#### Lysosomes

Due to low pH (*ca.* 5.0) and dozens of hydrolases, lysosomes are capable of degrading biological molecules, including foreign impurities and even necrotic organelles. Moreover, they also play important roles in autophagy, apoptosis and membrane repair. When lysosomes suffer from membrane rupture or dysfunctions, the internal hydrolases will be released, leading to cellular digestion, so lysosomes are regarded as a powerful target for cancer therapy. Compared with other organelles, nano-therapeutic agents can more easier enter lysosomes after experiencing endocytosis.^77^ Because positively charged agents are cannot penetrate the lysosomal membrane, pro-drugs bearing morpholine groups can aggregate in the lysosome.^68^

#### Endoplasmic reticulum (ER)

The ER is composed of a large biofilm system, which is involved in protein assembly, calcium homeostasis regulation and intracellular environmental stabilization. When the stimulation of the ER occurs, it not only blocks the protein synthesis but also triggers the apoptotic signaling pathway. Therefore, the ER is related to the occurrence of a variety of diseases. Meanwhile, it is also considered as one of the key aims of tumor-targeted therapy. So far, sulfamides^69^ and pardaxin (FAL) peptides^70^ have been widely used as good ligands for ER-targeted therapy.

## 2. Dual-targeted photothermal agents

DTPTAs have got great advances over the past few years, and many materials including noble metal materials, transition metal oxide/sulfide materials, carbon materials, cyanine dyes, and organic polymers as well as other materials that have been used for designing novel PTAs for tumor ablation (Table 2).

**Table 2:** PTAs for cancer therapy

PTAs	Irradiation^a^	Photothermal effect^a^	Targeted moieties	Specific sites or stimulation	Animal models	Ref.
Au nanoprisms	808 nm, 500 mW cm^−2^, 20 min	Δ*T* = ∼16 °C (30 μg mL^−1^)	TPE@Zn and AS1411 DNA aptamers	Cell membrane and nucleus	SGC-7901 human gastric carcinoma tumor model (significant inhibition)	63
AuNS^b^	808 nm, 1 W cm^−2^, 5 min	Δ*T* > 20 °C (4 μg mL^−1^)	HA and TPP	CD44 and mitochondrion	SCC-7 mouse squamous cell carcinoma tumor model (significant inhibition)	78
					MCF-7/ADR drug resistant tumor model (significant inhibition)	
GNS	808 nm, 1.74 W cm^−2^, 4 min	Δ*T* = ∼15 °C (3 μg mL^−1^ of Au)	HA and NLS	CD44 and nucleus	4T1 mouse breast cancer tumor model (significant inhibition)	79
					4T1 metastatic tumor model (reduced tumor metastasis)	
AuNRs^c^	808 nm, 4 W cm^−2^, 10 min	Increased to ∼55 °C (2 nM)	NLS and RGD	Nucleus and integrin α_v_β_3_	—	80
			MLS and RGD	Mitochondrion and integrin α_v_β_3_		
			RGD	Integrin α_v_β_3_		
GNR	808 nm, 2 W cm^−2^, 4 min	Increased to 43.5 °C (40 μg mL^−1^)	HA and RGD	CD44 and integrin α_v_β_3_	—	81
GNR	808 nm, 2 W cm^−2^, 10 min	Δ*T* = ∼30 °C (20 μg mL^−1^ of Au)	HA and FA	CD44 and FR	MCF-7 human breast cancer tumor model (complete elimination)	82
GNR	808 nm, 4 W cm^−2^, 10 min	Increased to ∼51.6 °C	cRGD^d^ and FA	Integrin α_v_β_3_ and FR	B16-F10 mouse melanoma tumor model (significant inhibition)	83
GNR	808 nm, 2 W cm^−2^, 10 min	Increased to ∼55 °C (20 μg mL^−1^ of Au)	Anti-HER2 antibody and HA	HER2 and CD44	MCF-7 human breast cancer tumor model (complete elimination without reoccurrence)	84
Au shell	808 nm, 1 W cm^−2^, 5 min	Δ*T* = ∼30 °C (1 mg mL^−1^)	Fe_3_O_4_ and FA	Magnetic field and FR	—	85
Au shell	808 nm, 1 W cm^−2^, 10 min	Δ*T* = ∼30 °C (40 μM)	Fe_3_O_4_ and MTX	Magnetic field and FR	4T1 mouse breast cancer tumor model (complete elimination)	86
AuNRs^c^	808 nm, 2 W cm^−2^, 4 min	Δ*T* > 20 °C (1 mg mL^−1^)	HA and SM	CD44 and acidity	MDA-MB-231 human breast cancer tumor model (almost complete suppression)	87
Au@Pt NPs	808 nm, 1.2 W cm^−2^, 10 min	Δ*T* = ∼65 °C (50 μg mL^−1^)	FA and TPP	FR and mitochondrion	—	88
CuS NPs	980 nm, 1.5 W cm^−2^, 10 min	Increased to 53 °C	RGD and TAT	Integrin α_v_β_3_ and nucleus	HeLa human cervical cancer tumor model (intratumoral/intravenous injection, complete obliteration)	89
					HeLa recurrent tumor model (no recurrence)	
WSSe	808 nm, 0.8 W cm^−2^, 10 min	Δ*T* = ∼42 °C (240 μg mL^−1^)	TPP and MCF-7 cell membrane	Mitochondrion and MCF-7 cell	MCF-7 human breast cancer tumor model (complete obliteration without recurrence)	90
Fe_3_O_4_	808 nm, 3 W cm^−2^, 500 s	Increased to ∼63 °C (100 μg mL^−1^ of IONP-20^e^)	Tf and TAT	TfR and nucleus	A549 human lung cancer tumor model (significant inhibition with a slow tumor growth)	91
SCDs	808 nm, 4 W cm^−2^, 10 min	Δ*T* = ∼33 °C (10 mg mL^−1^)	RGD and MLS	Integrin α_v_β_3_ and mitochondrion	—	67
MG^f^	808 nm, 6 W cm^−2^, 5 min	Increased to 50 °C (30 mg L^−1^)	Fe_3_O_4_ and IP^g^	Magnetic field and glioma	—	92
MGO	808 nm, 2.5 W cm^−2^, 3 min	Increased to ∼50 °C (1 mg mL^−1^)	Fe_3_O_4_ and CET	Magnetic field and EGFR	CT-26 murine colonic carcinoma cancer tumor model (significant inhibition)	93
ICG	808 nm, 1.54 W cm^−2^, 5 min	Increased to ∼61.5 °C (50 μg mL^−1^)	cRGD^d^ and FA	Integrin α_v_β_3_ and FR	—	94
ICG	808 nm, 2 W cm^−2^, 5 min	Increased to 55.2 °C (6.53 μg mL^−1^ of ICG)	Fe_3_O_4_ and HA	Magnetic field and CD44	U87MG human primary glioblastoma cancer tumor model (significant inhibition)	95
ICG	808 nm, 3 W cm^−2^, 100 s	Increased to 78.2 °C (40 μM)	RC-12 and PG-6	Integrin α_v_β_3_ and MMP-2 and MMP-9	—	37
ICG	808 nm, 1 W cm^−2^, 5 min	Δ*T* = ∼58 °C (60 μg mL^−1^)	FA and TPP	FR and mitochondrion	—	96
IR825 and carbon-derivatized FNPs^h^	808 nm, 2 W cm^−2^, 5 min	Increased to 65 °C (1 mg mL^−1^)	FA and TPP	FR and mitochondrion	—	97
IR825	808 nm, 0.8 W cm^−2^, 10 min	Δ*T* = ∼52 °C (500 μg mL^−1^)	Bevacizumab and IR825	VEGF and mitochondrion	C643 human anaplastic thyroid carcinoma tumor model (complete obliteration without recurrence)	34
*Bio* i *-PPh*_*3*_-PT	635 nm, 0.5 W cm^−2^, 10 min	Δ*T* = ∼25 °C (0.5 mM)	Biotin and TPP	BR and mitochondrion	4T1 mouse breast cancer tumor model (excellent inhibition)	55
ETP	650 nm, 0.5 W cm^−2^, 5 min	Δ*T* = ∼32.8 °C (100 μM + ALP 1 h)	Pi^j^ and TPP	ALP and mitochondrion	PC-3 human prostate cancer tumor mode (almost complete suppression)	98
PDA	808 nm, 0.8 W cm^−2^, 6 min	Δ*T* = ∼19 °C (0.2 mg mL^−1^)	Fe_3_O_4_ and TPP	Magnetic field and mitochondrion	B16-F10 mouse melanoma tumor model (significant inhibition with a slow tumor growth)	99
PDA and ICG	785 nm, 0.5 W cm^−2^, 500 s	Δ*T* = ∼10 °C (50 μg mL^−1^)	Tf and TPP	TfR and mitochondrion	A549 human lung cancer tumor model (complete obliteration)	100
FNPs-PDA	808 nm, 2 W cm^−2^, 5 min	Δ*T* = ∼25 °C	HA and TPP	CD44 and mitochondrion	—	101
MPDA	808 nm, 1 W cm^−2^, 5 min	Δ*T* = ∼38 °C (100 μg mL^−1^)	HA and MTX	CD44 and FR	4T1 mouse breast cancer tumor model (complete obliteration)	102
SP	808 nm, 0.8 W, 10 min	Δ*T* = ∼65 °C (10 μg mL^−1^)	FA and cRGD	FR and integrin α_v_β_3_	U87MG human primary glioblastoma cancer tumor model (promising suppression)	103
PB	808 nm, 2 W cm^−2^, 5 min	Δ*T* = ∼30 °C (1 mg mL^−1^)	Fe_3_O_4_ and HA	Magnetic field and CD44	S180 mouse sarcome tumor model (almost complete suppression)	104
V_2_C	1064 nm, 0.96 W cm^−2^, 10 min	Increased to 59.6 °C (400 μg mL^−1^)	RGD and TAT	Integrin α_v_β_3_ and nucleus	MCF-7 human breast cancer tumor model (significant inhibition without recurrence)	105
Aza-BODIPY	730 nm, 1 W cm^−2^, 12 min	Δ*T* = ∼28 °C (50 μg mL^−1^)	FA and TPP	FR and mitochondrion	HeLa human cervical cancer tumor model (complete suppression)	106
Melanin	808 nm, 1.5 W, 10 min	Δ*T* = ∼14 °C (100 μg mL^−1^)	RGD and PM^k^	Integrin α_v_β_3_ and tumor vasculature	MDA-MB-231/ADR human breast cancer drug resistant tumor model (significant inhibition without metastasis)	107

a Photothermal performance *in vitro*.

b AuNS: gold nanostars.

c Au NRs: gold nanorods.

d cRGD: cyclic RGD.

e INOP-20: 20 nm INOPs.

f MG: magnetic graphene.

g IP: interleukin-13-based peptide.

h FNPs: fluorescence NPs.

i *Bio*: biotin.

j Pi: phosphate ester group.

k PM: platelet membrane.

### 2.1. DTPTAs based on noble metal materials

Noble metal nanoparticles (NPs) are well-studied inorganic PTAs because of their excellent photothermal and optical properties. Due to localized surface plasmon resonance (LSPR), they exhibit great talents for absorbing laser to reach electron excited state and release heat *via* nonradiative decay.

Gold-based NPs, as excellent PTAs with intense LSPR, have got diverse applications *via* the modification of resonance wavelength. Researchers have developed a variety of gold nanostructures, such as gold nanorod (GNR), gold nanoshells, gold nanoprisms and gold nanostars (GNS).

Although Au NPs can passively accumulate in tumors *via* enhanced permeability and retention effect, the efficiency of material utilization is low. Due to the convenience of surface modification, Au NPs can be functionalized with active targeting ligands (*e.g.*, antibodies, aptamers and peptides) to realize selective accumulation in tumor tissues.

**Fig. 1:**
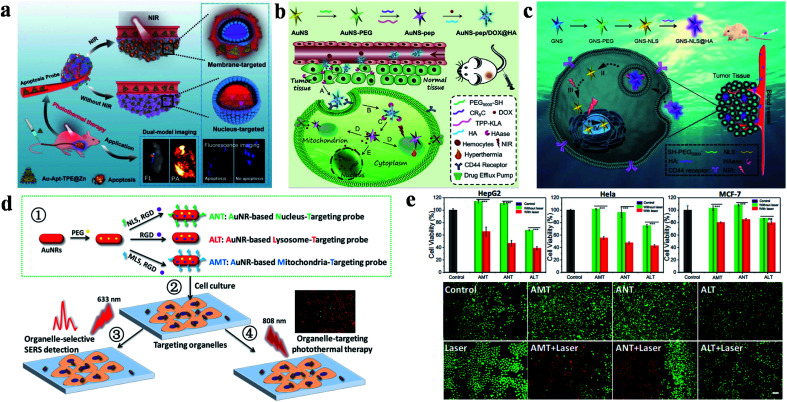
(a) Schematic diagram of Au-Apt-TPE@Zn for dual-model imaging and dual-targeted cancer PTT. Reproduced from ref. 63 with permission from Zhang *et al.* (b) Schematic diagram of the preparation and targeting principles of AuNS-pep/DOX@HA. Reproduced from ref. 78 with permission from Elsevier Ltd, copyright 2016. (c) Schematic diagram of the preparation and targeting principles of the cellular/intranuclear dual-targeting nanoplatform GNS-NLS@HA. Reproduced from ref. 79 with permission from the Royal Society of Chemistry, copyright 2018. (d) Schematic diagram of the preparation and application of AuNR-based nanoprobes. (e) The effect of AuNR-based nanoprobes on cell viability. Adapted from ref. 80 with permission from the American Chemical Society, copyright 2018.

Recently, Sun's group successfully prepared and characterized dual-targeted gold nanoplatforms (Au-Apt-TPE@Zn, Apt: aptamer), which were used to identify early apoptotic cells and perform accurate PTT for tumors (Fig. 1a).^63^ In their study, gold nanoprisms were coupled with phenanthroline derivatives (functionalized with tetraphenylethene (TPE)), and further stabilized with target peptide aptamers through Au–S bonds to obtain Au-Apt-TPE. The remaining nitrogen atoms of Au-Apt-TPE effectively chelated with Zn^2+^ to synthesize Au-Apt-TPE@Zn. Moreover, Au-Apt-TPE@Zn selectively recognized early apoptotic cells and bound cell membrane. Connected to AS1411 DNA aptamers, Au-Apt-TPE@Zn specifically targeted the nucleus. The dual-targeted therapeutic agent for cell membrane and nuclei showed prominent photothermal conversion efficiency (PCE) and allowed the nanoplatform to achieve accurate treatment for tumor inhibition.

In addition, a dual-targeted photothermal material for targeting tumor cells and mitochondria was also studied by Zhang's group (Fig. 1b).^78^ AuNS-pep/DOX@HA (pep: peptide) was composed of cationic peptide R8, TPP modified α-helical pro-apoptotic peptide (TPP-KLA), doxorubicin (DOX) and HA. The nanoplatform was internalized into tumor cells through CD44 receptor-mediated recognition. TPP-KLA was used to specifically insert and destroy mitochondrial membranes, and then induce dysfunction of the mitochondria and cause mitochondrial-dependent apoptosis. *In vitro* and *in vivo* experiments showed that AuNS-pep/DOX@HA presented prominent non-resistant or resistant tumor inhibition. In 2018, the authors also designed a cellular/intranuclear dual-targeted nanoplatform for tumor therapy *via* PTT (Fig. 1c).^79^ NLS peptides were used to modify GNS to give GNS-NLS for targeting the nucleus. Subsequently, HA was coated on the surface of GNS-NLS (GNS-NLS@HA) through electrostatic interactions, which then bound with CD44 to enhance internalization and then expose NLS after degradation by hyaluronidase (HAase). GNS-NLS@HA showed high photothermal conversion (Δ*T* = ∼15 °C, 808 nm, 1.74 W cm^−2^, 4 min) and prominent tumor suppression efficiency.

Moreover, Xu and co-workers explored the therapeutic effects of GNRs in different subcellular organelles with the modification of specific targeting peptides and RGD peptides (Fig. 1d).^80^ Surface-enhanced Raman scattering spectroscopy promoted AuNRs to achieve super-resolution imaging of biomolecules and monitor the position of therapeutic agents. Under the same experimental conditions, AMT (AuNRs modified with MLS and RGD) or NLS (AuNRs modified with NLS and RGD) have better tumor killing effect than ALT (AuNRs only modified with RGD) (Fig. 1e).

**Fig. 2:**
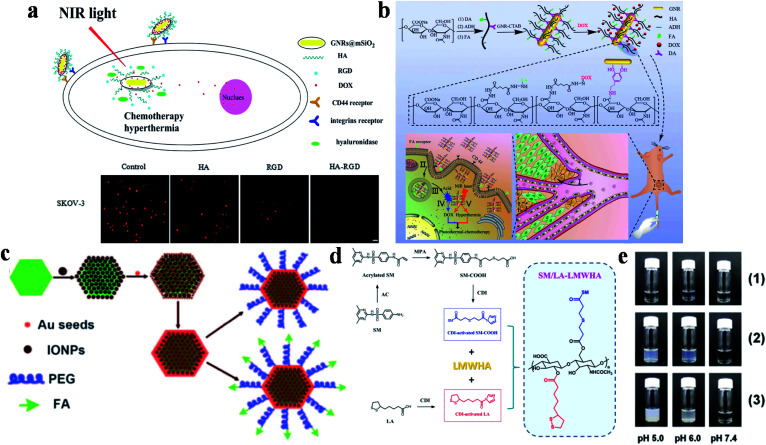
(a) Schematic diagram of DOX-GNRs@mSiO_2_-HA-RGD for targeted therapy and cell uptake studies of DOX-GNRs@mSiO_2_-HA-RGD with different inhibitors (HA, RGD and HA-RGD). Adapted from ref. 81 with permission from Elsevier B.V., copyright 2017. (b) Schematic diagram of the preparation and targeting principles of GNRs-HA-FA-DOX. Adapted from ref. 82 with permission from the American Chemical Society, copyright 2017. (c) Schematic diagram of the preparation method of MFNPs. Reproduced from ref. 85 with permission from Wiley-VCH Verlag GmbH & Co. KGaA, Weinheim, copyright 2011. (d) Schematic diagram of the synthesis of SM/LA-LMWHA. (e) The effects of different pH on (1) LMWHA, (2) SD/LA-LMWHA, and (3) SM/LA-LMWHA. Reproduced from ref. 87 with permission from Elsevier Ltd, copyright 2019.

Another effective dual-targeted model is the combination of two different tumor cell-targeted ligands such as RGD, HA and FA. Recently, Xu and co-workers reported a mesoporous silica (mSiO_2_)-coated nanoplatform (DOX-GNRs@mSiO_2_-HA-RGD) by combining HA, RGD and DOX (Fig. 2a).^81^ Cell uptake studies proved that the internalization of the dual-targeted DOX-GNRs@mSiO_2_-HA-RGD was more effective than single-targeted and untargeted therapeutic agents. In addition, DOX-GNRs@mSiO_2_-HA-RGD showed excellent photothermal conversion properties, with a rapid increase to 43.5 °C (808 nm, 2.00 W cm^−2^, 4 min). Yao and co-workers designed a pH and near-infrared (NIR) dual-responsive drug release nanoplatform for the cooperative chemo-photothermal therapy of breast cancer, which was prepared by combining GNR, FA, HA, dopamine, adipic acid dihydrazide and DOX (Fig. 2b).^82^ Compared with the non-FA-modified NPs, the dual-responsive nanoplatform was more efficient to deliver GNR and DOX to MCF-7 cells. Moreover, tumor cells were killed more effectively through inducing apoptosis under NIR irradiation. Recently, Li and co-workers also studied the therapeutic effect of DOX-loaded FA/RGD dual-targeted GNR (denoted as FA/RGD-DOX-*hz*-GNRs, *hz*: a pH-sensitive hydrazone) for B16-F10 xenograft tumors.^83^*In vivo* experiments showed that this dual-targeted nanoplatform exhibited excellent tumor therapeutic effects *via* the synergistic cooperation of DOX-induced apoptosis, heat induced necrosis and angiogenesis inhibition. Other than that, human epidermal growth factor receptor 2 (HER2) is also an important targeting site for tumor therapy, which is over-expressed in multiple tumors. Conjugated with HER2, a pH, GSH and HAase triple-responsive nanoplatform GNR-HA^-ALA/Cy7.5^-HER2 (ALA: 5-aminolevulinic acid) was reported by Yao and co-workers.^84^*In vivo* experiments illustrated that tumor tissues could be completely eliminated without obvious side effects. This nanoplatform provided a potential strategy for HER2/CD44 targeted breast cancer PDT/PTT.

Furthermore, dual-targeted therapy can also be achieved *via* simultaneously targeting the tumor environment and tumor cells. Liu's group modified ultrasmall superparamagnetic IONPs onto NaYF_4_-based upconversion NPs, and then coated a thin gold shell through seed-induced reduction growth, on which poly(ethylene glycol) (PEG) were anchored as linkers to conjugate FA to prepare the multifunctional platform (denoted as FA-PEG-MFNP) (Fig. 2c).^85^ FA-PEG-MFNP could achieve dual-modal targeting capability mediated by magnetic field and FR with the monitoring of upconversion luminescence and MR imaging. After that, Zhang's group constructed a MTX-Fe_3_O_4_-gold shell (MFG) nano-system hybridized with LPM lipoic acid (LA)-PEG-MTX (LPM) to obtain MFG-LPM NPs for dual-targeted chemo-photothermal therapy.^86^ MTX has a similar structure to FA, which can be used for cancer therapy as both targeted ligand and chemotherapeutic drug. This dual-targeted MFG-LPM had excellent healing talent that cured all tumor-bearing mice in a short time, while mice in none-magnetic group needed a second injection therapy.

The acidic microenvironment of tumor tissues has also been used for dual-targeted cancer therapy. Lee's group combined low molecular weight hyaluronic acid (LMWHA), the pH-sensitive group sulfamethazine (SM) and GNR to synthesize dual-targeted therapeutic platforms from ligand-mediated cell uptake and acid-induced aggregation (Fig. 2d).^87^ When the pH was lower than 7.4, the de-ionization of SM in AuNRs@SM/LA-LMWHA improved the surface hydrophobicity, resulting in particle aggregation and gradually increasing size (Fig. 2e). And then, the NPs were internalized into tumor cells through CD44 receptor-mediated recognition for tumor therapy.

Other noble metals (*e.g.*, platinum) are also used as DTPTAs for enhanced tumor therapy by taking the advantages of photothermal stability and certain catalytic ability. In 2017, Lin's group developed Au@Pt NPs modified with a cell-targeting ligand FA and a mitochondria-targeting group TPP for PDT/PTT synergistic therapy.^88^ In this nanoplatform, the excellent catalytic activity of Pt NPs could enhance the peroxidase-like catalysis of H_2_O_2_ to improve the efficacy of PDT.

### 2.2. DTPTAs based on transition metal oxide/sulfide materials

**Fig. 3:**
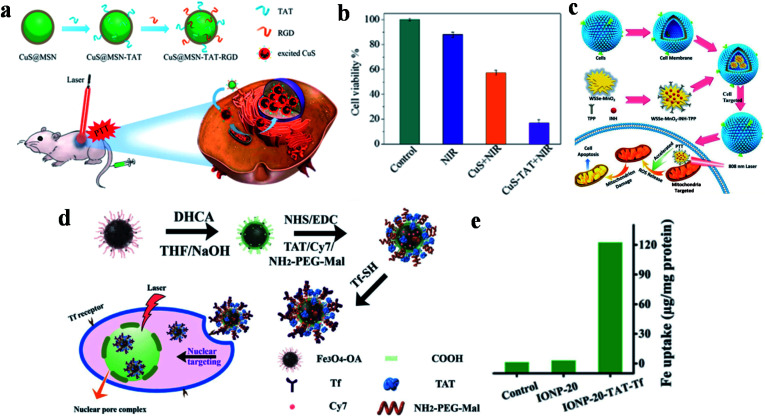
(a) Schematic diagram of the preparation and targeting principles of CuS@MSN-TAT-RGD NPs. (b) The effect of different treatment conditions on cell viability. Reproduced from ref. 89 with permission from the American Chemical Society, copyright 2018. (c) Schematic diagram of the preparation and treatment principles of WSSe/MnO_2_-INH-TPP@CM. Reproduced from ref. 90 with permission from Wiley-VCH Verlag GmbH & Co. KGaA, Weinheim, copyright 2019. (d) Schematic diagram of the preparation and targeting principles of IONP-TAT-Tf. (e) Nuclear iron elemental quantification of IONP-20 and IONP-20-TAT-Tf by A549 cells. Reproduced from ref. 91 with permission from Wiley-VCH Verlag GmbH & Co. KGaA, Weinheim, copyright 2017.

Although noble metal NPs such as gold and platinum have shown great potential applications in the field of tumor photothermal treatment, the high cost, difficult biodegradation and toxicity during long-term metabolic processes in the body have restricted further research and clinical practice application. Transition metal oxide/sulfide NPs as another type of inorganic PTAs exhibit strong NIR-absorption, low cost and high PCE, which has attracted much attention in PTT for tumor tissues. Because of these advantages, copper sulfide (CuS) NPs are widely used in the treatment of multifarious diseases such as cancer,^108,109^ atherosclerosis^110^ and diabetes,^111^ and show excellent biological applications based on low cytotoxicity and controllable morphology. Tang and co-workers developed a nucleus-targeted PTT strategy based on CuS NPs to achieve intra-nuclear PTT and effectively prevent local cancer recurrence (Fig. 3a).^89^ Through the surface modification of mesoporous silica coated CuS (CuS@MSN) by RGD and TAT peptides, CuS@MSN-TAT-RGD NPs directly targeted tumor cells and further entered the nucleus. After irradiation with a 980 nm NIR laser, the endonuclear genetic materials were destroyed by hyperthermia, resulting in cell necrosis. Under 980 nm laser irradiation for 5 minutes, the cell killing rate of the NPs with targeting ability was 84%, while that of non-targeting NPs was 42% (Fig. 3b). This PTT platform, with the capability of targeting nucleus, provides powerful possibilities for tumor ablation and anti-recurrence.

WSSe nanosheets were also used in dual-targeted PTT for tumors by Zhang's group *via* the preparation of a WSSe/MnO_2_-INH nanocomposite (Fig. 3c).^90^ In this study, Mn^2+^ generated by the degradation of MnO_2_ was used to catalyze the isoniazid (INH) to obtain hydroxyl radicals (˙OH) for achieving PTT and PDT anticancer treatment. WSSe/MnO_2_-INH-TPP@CM (CM: cell membrane) was coated with cancer cell membrane extracted from the breast cell line MCF-7, and then it was modified with the mitochondrial-targeted group TPP to realize dual-targeted tumor therapy. WSSe/MnO_2_-INH-TPP@CM showed good anti-cancer effects both *in vivo* and *in vitro*.

Moreover, Yang and co-workers developed nucleus-targeted multifunctional magnetic NPs (IONP-20-TAT-Tf) bearing Tf and TAT peptides (Fig. 3d).^91^ The conjugated monodisperse magnetic NPs exerted considerable photothermal stability and high PCE (∼37%). Quantitative analysis showed that the accumulation of multifunctional IONP in the nucleus was 45-fold higher than that without TAT modification (Fig. 3e). During *in vivo* experiments, the composite NPs effectively inhibited xenografted tumors irradiated by NIR lasers, and are expected to be used in cancer treatment.

### 2.3. DTPTAs based on carbon materials

**Fig. 4:**
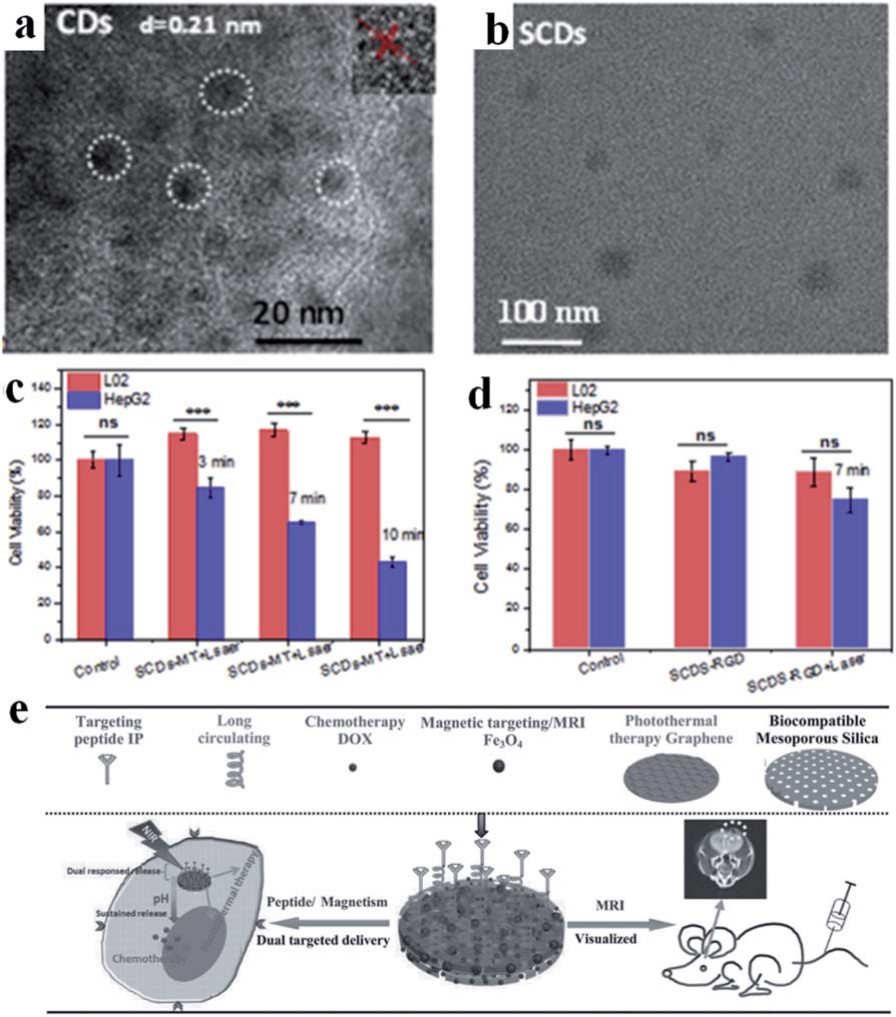
TEM images of (a) CDs and (b) SCDs. Cell viabilities of L02 cells and HepG2 cells treated with (c) SCDs-MT and (d) SCDs-RGD and irradiated for 7 min. Adapted from ref. 67 with permission from Elsevier Ltd, copyright 2019. (e) Schematic diagram of the preparation and application of MGMSPID. Reproduced from ref. 92 with permission from Wiley-VCH Verlag GmbH & Co. KGaA, Weinheim, copyright 2013.

Carbon-based nanomaterials have great potential applications in the field of tumor PTT. Graphene oxide, carbon dots and other carbon-based materials have extensive optical absorption and reasonable photothermal properties, which has aroused widespread interest. Carbon nanodots have outstanding characteristics, such as high photostability, low toxicity, high solubility, low cost and good biocompatibility. However, the low efficiency of the absorption in the visible to NIR window limits their application in light-sensitive cancer treatment strategies and *in vivo* imaging. Xu's group prepared a new type of super carbon dots (SCDs) (about 20 nm) with broad absorption from the visible light to NIR region.^67^ The carbon dots (CDs) possess a high PCE for PTT, and can be functionalized with dual-targeted ligands (RGD and MLS) for cancer cells and mitochondria. The SCDs were constructed *via* the self-assembly of small sized CDs (around 5 nm) in an acidic environment, which could be located precisely in the mitochondria of cancer cells for cancer therapy (Fig. 4a and b). The experimental results showed that the difference in the survival rate between the tumor and normal cells was as high as 70%, reflecting the high specificity and selectivity of these SCDs (Fig. 4c and d).

Due to its large specific surface area, excellent electrical properties and optical properties, graphene has attracted extensive attention in many fields in recent years. Graphene oxide can be obtained *via* the chemical oxidation of graphene, which has a good PCE for PTT. Kong's group successfully prepared multifunctional magnetic graphene as a nanomedical platform (denoted as MGMSPID) for MRI guided chemo-photothermal glioma treatment (Fig. 4e).^92^ This photothermal agent could be easily prepared *via* a PEGylated method and then modified with an interleukin-13-based peptide (IP). In addition, the MGMSPID system showed thermal stimulation, pH-response and sustained release properties. All these features provide a powerful multi-functional therapeutic platform for visual glioma treatment. Chen's group also developed a pH-sensitive dual-targeted magnetic nanocarrier for chemo-phototherapy in cancer treatment.^93^ The authors prepared magnetic graphene oxide (MGO) *via* chemical coprecipitation, and then modified MGO with PEG and CET to obtain MGO-PEG-CET. MGO-PEG-CET was used for magnetic and receptor-mediated dual-targeted synergistic treatment to delivery DOX to EGFR over-expressed tumor cells. *In vitro* cytotoxicity tests showed that the half-maximum inhibitory concentration (IC_50_) value of the dual-targeted nanoplatform MGO-PEG-CET/DOX on CT-26 cells was 1.48 μg mL^−1^, which was lower than the group of MGO-PEG/DOX (2.64 μg mL^−1^).

### 2.4. DTPTAs based on cyanine dyes

Compared with inorganic PTAs, organic PTAs have the inherent advantages of biocompatibility and degradability, among which cyanine dyes are the most representative agents, which have widespread applications. Indocyanine green (ICG), one of the cyanine dyes, has drawn a lot of attention due to its great biosafety, and has been approved by the Food and Drug Administration for clinical fluorescence imaging.^112^ ICG also shows great properties for tumor PTT, PDT and fluorescence imaging, with satisfying water solubility and potential mitochondrial targeting, as well as splendid PCE properties. However, since ICG is easily cleared *in vivo* and has a short cycle time, it is usually administered *via* a delivery system. Obviously, a dual-targeted drug delivery system is an ideal strategy to carry ICG to tumor tissues for imaging and therapy. In 2018, Wang *et al.* developed thermo-sensitive NPs to drive ICG, photosensitizer chlorin e6 (Ce6) and chemotherapy drug cisplatin into cancer cells with the help of tumor recognition groups.^94^ Targeted FA molecules and cRGD peptides were covalently anchored to different amphiphilic polymers and the modified polymers self-assembled to form NPs to enhance the active targeting properties.

**Fig. 5:**
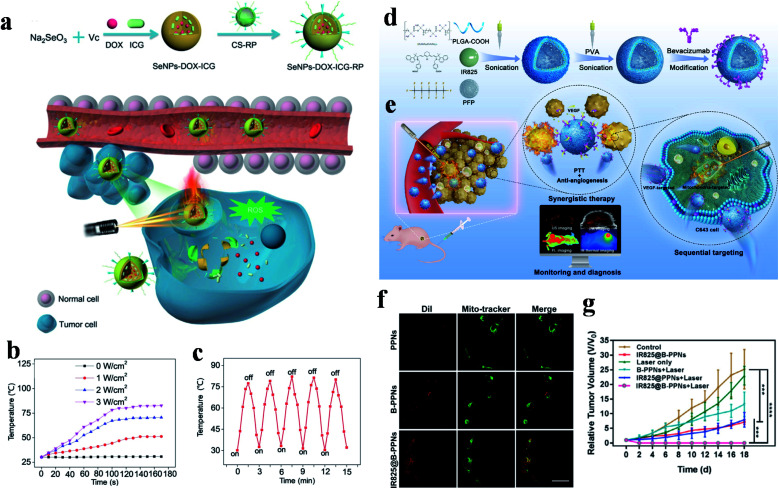
(a) Preparation and delivery mechanisms of SeNPs-DOX-ICG-RP with NIR laser irradiation. (b) Temperature changes of SeNPs-DOX-ICG-RP under different irradiation powers. (c) Photothermal stability experiment. Adapted from ref. 37 with permission from Wiley-VCH Verlag GmbH & Co. KGaA, Weinheim, copyright 2018. (d) Synthesis and (e) sequential targeting process of IR825@B-PPNs with monitoring and diagnosis. (f) CLSM images of mitochondrial localization in C643 cells. The scale bar is 20 μm. (g) Tumor growth curves after treatment with different therapies. Adapted from ref. 34 with permission from Acta Materialia Inc, published by Elsevier Ltd, copyright 2019.

This intelligent drug release realized synergistic treatment and ablated tumors under NIR irradiation. To improve the cancer-targeted efficiency, Anilkumar *et al.* constructed dual targeted magnetic photosensitive liposomes (MPLs) to deliver ICG to tumor tissues driven by a magnetic field and to internalize drugs into cells *via* the combination of HA and CD44.^95^ In this study, citric acid-coated Fe_3_O_4_ magnetic NPs (CMNs) and ICG molecules were loaded in MPLs *via* a solvent evaporation/hydration technique, and then HA-PEG was connected by electrostatic interaction triggered self-assembly to fabricate HA-PEG-MPLs. These HA-PEG-MPLs particles not only achieved tumor aggregation enhanced therapy, but also increased the PCE (Δ*T* = ∼30 °C, 808 nm, 2.0 W cm^−2^, 5 min) *via* the combined influence of ICG and CMNs. Therefore, HA-PEG-MPLs showed superior inhibiting ability in a xenograft tumor model. Fang *et al.* constructed biocompatible selenium NPs (SeNPs) to deliver DOX and ICG to tumor cells (Fig. 5a).^37^ In order to assure agents arrived in tumor cells successfully, chitosan was chosen as a linker to conjugate with RGD-derived RC-12 peptide and MMP-recognized PG-6 peptide to give SeNPs-DOX-ICG-RP. The internalization of tumor cells triggered by endocytosis offers great possibilities of accurate treatment.

Besides this, the Se nanosystems showed amazing PCEs and temperatures that could increase to 78.2 °C (808 nm, 3.0 W cm^−2^, 100 s), and the excellent stability was also certified *via* a laser turn on/off experiment five times (Fig. 5b and c).

Wu *et al.* constructed a tumor and mitochondria dual-targeted nanocarrier for mitochondrial locating PTT and chemotherapy.^96^ In this case, FA and TPP were respectively connected to amphiphilic polymer DSPE-PEG, and the resulting polymers were mixed and self-assembled to form nanovesicles for encapsulating ICG and DOX. The vesicles displayed great colloid stability and released DOX *via* the rapid temperature rise (Δ*T* = ∼58 °C, 808 nm, 1.0 W cm^−2^, 5 min) induced by NIR irradiation. Importantly, combined with FA and TPP, these nanoagents showed powerful mitochondria locating capability (*p* up to 0.84) and enhanced cytotoxicity, with a sharply reduced MCF-7 cell survival rate of as low as 3.8%.

IR 825 is a kind of heptamethine type NIR dye with perfect photothermal conversion performance. Despite possessing a similar structure to ICG, IR 825 has limited applications in the theranostic field due to low water solubility, so it is usually delivered to tumor tissue *via* a micelle encapsulation technique. In 2017, Kang *et al.* described a FA and TPP modified polymeric core–shell therapeutic agent for specifically transmitting IR 825 and 3-bromopyruvate (BP) to tumor mitochondria.^97^ PEG grafted poly(dimethyl aminoethyl methacrylate) (PEG-*g*-PDMA) was connected with catechol and carbonized with sulfuric acid to obtain the core, and the shell was composed of PEG-*g*-PDMA, tumor-targeted FA, mitochondria-targeted TPP, IR 825 and phenylboric acid, which reacted with catechol to achieve core–shell binding. This therapeutic agent damaged tumor mitochondria and induced cell death triggered by hyperpyrexia (up to 65 °C) and the glycolysis inhibition of BP under NIR irradiation (808 nm, 2.0 W cm^−2^, 5 min).

Since the IR 825 molecules have lipophilicity cationic structure for mitochondrial aggregation, they are also used for mitochondrial-targeted therapy. Based on this feature, Wang *et al.* engineered a targeted theranostic agent (IR825@B-PPNs) for anaplastic thyroid carcinoma oncotherapy (Fig. 5d and e).^34^ IR 825 and perfluoropentane (PFP, an ultrasound contrast agent) were loaded in poly(lactic-*co*-glycolic acid) (PLGA) to form IR825@PPNs, and then, polyvinyl alcohol (PVA) and bevacizumab antibodies were modified (IR825@B-PPNs) in order to promote tumor aggregation. The conjugated bevacizumab specifically recognized VEGF and blocked the combination of VEGF and receptors. With the monitoring and diagnosis of the photoacoustic, fluorescence, and ultrasonic multi-mode imaging, tumor and mitochondrial aggregation enhanced the thermal damage of IR 825 to tumor cells. The well-merged fluorescence signals of IR825@B-PPNs and Mito-Tracker indicated that the theranostic agent had a tendency for mitochondrial localization and *in vivo* experiments realized the complete curing of a C643 tumor (Fig. 5f and g).

**Fig. 6:**
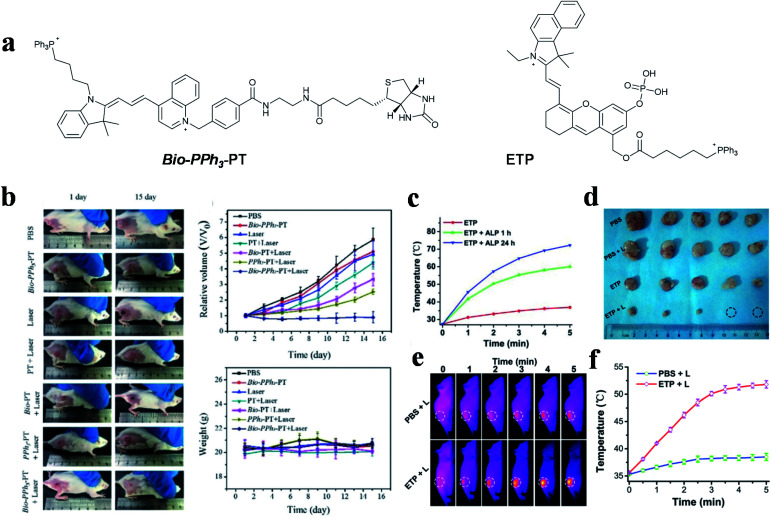
(a) Dual-targeted molecules *Bio-PPh*_*3*_-PT and ETP. (b) Therapeutic effect under different treatments. Adapted from ref. 55 with permission from Wiley-VCH Verlag GmbH & Co. KGaA, Weinheim, copyright 2019. (c) Temperature change curves of ETP treated with different concentrations of ALP under NIR laser irradiation. (d) Tumor photographs of mice. (e) Thermal imaging and (f) quantitative curves of PC-3 tumor-bearing mice. Adapted from ref. 98 with permission from the Royal Society of Chemistry, copyright 2019.

Other cyanine dyes also make a great contribution to dual-targeted PTT. In 2019, Tang and co-workers designed a novel small molecule (*Bio-PPh*_*3*_-PT) for highly effective and selective tumor mitochondrial PTT (Fig. 6a left).^55^ In this study, quinoline and indole blocks were connected *via* continuous double bonds to obtain a solid compound that possesses the ability of photothermal conversion, and then conjugated it with biotin and TPP for targeting tumors and mitochondria. The DTPTAs showed a superb PCE (37.8%) with a temperature change of ∼25 °C (635 nm, 0.5 W cm^−2^, 5 min). Biotin was used to distinguish diseased tissue from normal tissue and TPP functioned as an important targeting group for locating in mitochondria. The dual-targeted photothermal material can cause irreversible damage at a light power as low as 0.5 W cm^−2^. Besides this, during the treatment of tumor-bearing mice, apparent inhibition occurred in the dual-targeted group, while the therapeutic effect in the non-targeted group was negligible (Fig. 6b).

The photothermal conversion capability of small molecule PTAs was regulated by the electrical changes initiated from the regulation of specific substances in tumor cells.^113^ An aggregation-enhanced PTT probe (termed as ETP) was developed by Yao *et al.* for mitochondria-targeted imaging and therapy,^98^ which was activated by alkaline phosphatase (ALP) (Fig. 6a right). A phosphate ester group was introduced into the body of the probe for inhibiting molecular activity. After hydrolysis by ALP, which is overexpressed in prostate cancer, molecule absorption occurred with a large redshift, to allow active FL and photoacoustic imaging. Simultaneously, the photothermal performance and therapeutic effect were enhanced *via* molecular aggregation and mitochondria-targeting properties. The probes showed a 32.8 °C temperature change in *in vitro* experiment after incubation with ALP for 24 h, while the group without incubation only changed by 9.5 °C (650 nm, 0.5 W cm^−2^, 5 min) (Fig. 6c). Thermal imaging showed that the tumor temperature increased to over 50 °C, which indirectly illustrated the effective tumor healing ability of the activated DTPTAs (Fig. 6d–f).

### 2.5. DTPTAs based on organic polymers

**Fig. 7:**
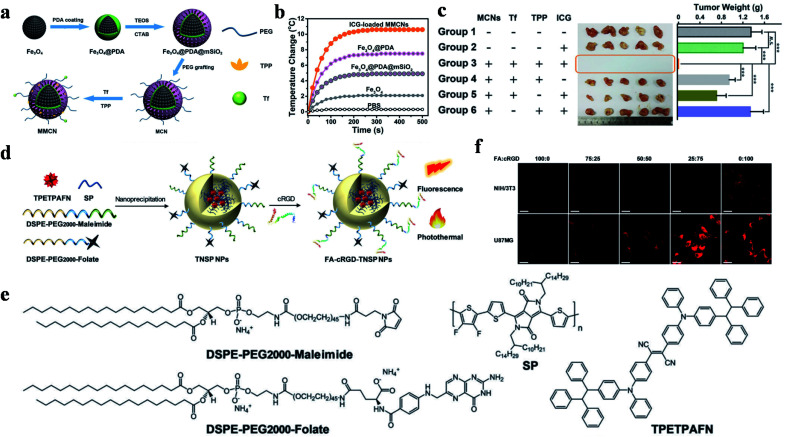
(a) Schematic illustration of the preparation of magnetic/mitochondria-targeting composite NPs. (b) The photothermal response curves of different NP solutions with an NIR laser. (c) Tumor photograph (middle) and quantitative weights (right) of the different treatment groups; reproduced from ref. 100 with permission from Wiley-VCH Verlag GmbH & Co. KGaA, Weinheim, copyright 2016. (d) Schematic diagram of NP preparation. (e) Chemical structures of the compositions. (f) CLSM localization images in NIH/3T3 normal cells and U87MG GBM cells incubated with different proportions of targeting ligand modified NPs. Scale bars: 50 μm. Adapted from ref. 103 with permission from the Royal Society of Chemistry, copyright 2019.

Organic polymers have better light stability than small molecules, which have attracted scientists' interest for PTT. Among these polymers, polydopamine (PDA) plays a crucial role in PTT due to it being in low cost and easy to synthesize. It is worth mentioning that PDA as a natural biodegradable polymer has been widely used in targeted PTT. Mitochondria-targeted PTAs were demonstrated by Wang *et al.* using magnetic materials and TPP as targeting ligands.^99^ Magnetic Fe_3_O_4_ NPs were coated with PDA to form core–shell structures, on which TPP and GSH responsive PEG were grafted. At last, DOX was further adsorbed on the surface *via* π–π stacking to give the desired therapeutic agent Fe@PDA-TPP/SS/DOX. This multistage targeted therapeutic agent was guided by an external magnetic field to reach tumor tissues, and experienced lysosome escape and GSH mediated degradation to expose TPP for mitochondrial aggregation. Under low power density irradiation, Fe@PDA-TPP/SS/DOX exerted an excellent temperature increase of 19 °C within 6 minutes (808 nm, 0.8 W cm^−2^) and released DOX to destroy mitochondrial DNA. Analogously, Guo *et al.* also constructed a Fe_3_O_4_@PDA@mSiO_2_ core–shell material for targeted PTT (Fig. 7a).^100^ In this study, tumor-identified Tf and mitochondria-targeted TPP were anchored on the surface of Fe_3_O_4_@PDA@mSiO_2_ materials to enhance their transport to tumor subcellular organelles. In addition, ICG molecules were loaded in the outermost mSiO_2_ layer to improve photothermal conversion and produce ROS. This composite material showed a considerable capacity of heat production (Δ*T* = ∼10 °C, 785 nm, 0.5 W cm^−2^, 500 s) under a low dose of light radiation and outstanding tumor ablation with complete healing efficacy (Fig. 7b and c).

After carbonizing them with concentrated sulfuric acid, PDA ramified fluorescent carbon NPs (FNPs-PDA) were synthesized by Park and co-workers.^101^ FNPs-PDA, acting as a PTA and imaging agent, underwent a boracic acid mediated esterification reaction to give it a HA shell, which was then modified with TPP and β-cyclodextrin (β-CD) to obtain the nanocomposite drug FNPs-PDA@HA-TPP-CD-PTX. When the therapeutic agent arrived at the tumor tissues, the combination of HA and CD44 promoted endocytosis and TPP triggered mitochondrial aggregation after lysosome escape. The low pH in the lysosome not only promoted the heat production of FNPs-PDA@HA-TPP-CD-PTX, but also facilitated the release of paclitaxel (PTX) in the β-CD hydrophobic cavity for multimodal therapy.

Zhu and co-workers reported magnetic polydopamine (MPDA), which was loaded with DOX and then coated with HA-MTX.^102^ In this study, the magnetic Fe_3_O_4_ NPs guided tumor aggregation *in vitro* in a magnetic field, while HA as an active targeting ligand enhanced CD44 mediated endocytosis. Besides this, MTX played the dual roles of chemotherapeutic drug and ligand to FR. *In vitro* experiments showed that tumor cells could be efficiently killed by the synergistic treatment of PTT and chemotherapy based on a high PCE (Δ*T* = ∼38 °C, 808 nm, 1.0 W cm^−2^, 5 min).

Semiconducting polymers (SP) are chain-like macromolecular materials with semiconductor properties. Due to their advantages of long wavelength absorption and efficient PCE, SP have been widely studied for PTT and photoacoustic imaging.^114–120^ Cai *et al.* used SP as a photothermal agent for glioblastoma multiforme (GBM) ablation, and investigated the influences of two targeted ligands in different ratios on tumor internalization (Fig. 7d and e).^103^ SP and aggregation-induced emission molecules were loaded in DSPE-PEG_2000_, meanwhile FA and RGD were conjugated on the surface to achieve specific tumor accumulation. *In vitro* and *in vivo* experiments showed that the SP exhibited a perfect light absorption and PCE in the NIR region, which resulted in a rapid temperature rise of ∼65 °C after 10 min of exposure (808 nm, 0.8 W). Moreover, NPs modified with FA : RGD (25 : 75) were proven to have superior endocytosis ability (Fig. 7f).

### 2.6. DTPTAs based on other materials

Prussian blue (PB) as an ancient pigment has also been used as a PTA to treat tumor in recent years. Taking advantage of the absorption of PB in the NIR region, a multifunctional PTA was designed and synthesized by Du *et al.* for dual-targeted PTT.^104^ In their study, PB NPs were designed as a kind of PTAs, while magnetic Fe_3_O_4_ NPs were adsorbed on the surface of upconversion NPs as magnetic targeting ligands. In addition, PEG-HA was then modified on the outermost layer to enhance the water solubility and active targeting of the material. Upon being exposed to NIR light, the composite materials rapidly increased in temperature from 20 to 50 °C in 5 min (808 nm, 2.0 W cm^−2^). *In vivo* experiments showed that they exhibited around four-fold higher aggregation in tumor than control groups without targeting ligands.

The limited penetration depth of light and the thermal resistance caused by heat shock proteins significantly restricts the therapeutic efficiency of PTT. To solve these problems, Zhang's group developed a nucleus-targeting strategy for low-temperature PTT in the NIR-II region.^105^ The authors first modified V_2_C quantum dots with a nucleus-targeting TAT peptide (V_2_C-TAT), and then wrapped the V_2_C-TAT quantum dots into an endogenous exosome (Ex). Then, RGD was connected to quantum dots to obtain a cancer cell membrane and nucleus dual-targeted system (V_2_C-PEG-TAT@Ex-RGD). This V_2_C-PEG-TAT@Ex-RGD system exhibited good biocompatibility, long circulation time, endosome escape and amazing PCE in the NIR-II region. Furthermore, this type of PTA can target cells and enter the nucleus to achieve low-temperature PTT with good tumor destruction efficiency.

Boron dipyrromethene (BODIPY) has also been extensively studied due to its prominent extinction coefficient, photostability, photothermal and photodynamic capabilities. In 2013, Chen *et al.* described an aza-BODIPY derivative (termed as MeOABBr) for multimodal imaging and phototherapy.^106^ The designed MeOABBr molecule exhibited a high singlet oxygen quantum yield (up to 84%) and excellent PCE (up to 40%), which made it possible to achieve tumor suppression by phototherapy. After mixing with PEG-NH_2_, PEG-TPP and PEG-FA, the molecules formed NPs *via* self-assembly for tumor elimination. *In vivo* experiments showed that the synergistic effect of PTT and PDT inhibited and eliminated HeLa tumors, and presented little damage to normal tissues and organs.

Melanin, a natural polymer that exists in many organisms, has been applied as a biocompatible therapeutic agent due to its excellent NIR light absorption and PCE. Wang and co-workers encapsulated melanin NPs (MNPs) and DOX into RGD-modified platelet vesicles to create a dual-targeted drug delivery system, which was used for treating multidrug resistant cancer and inhibiting metastasis.^107^*In vivo* experiments illustrated that the growth and metastasis of resistant breast cancer were efficiently inhibited by DOX and the MNPs (Δ*T* = ∼14 °C, 808 nm, 1.5 W, 10 min).

## 3. Conclusions

In recent years, PTT based on dual-targeted ligands has attracted extensive attention, and great advances have been made in this field. In this perspective, typical targeting strategies can be divided into three parts, “extracellular targets”, “intracellular targets”, and “subcellular targets” according to the recognizing position. Compared with traditional PTAs, DTPTAs have the great advantage of being able to precisely target cancer cells or organelles. First, dual-targeted PTT shows better selectivity and decreased side effects to the surrounding tissues. Second, dual-targeted therapy makes tumor cells uptake DTPTAs more easily to improve therapy efficiency.

Although great efforts have been made to develop efficient DTPTAs, there are still some challenging problems to be resolved in the future. (a) Photosensitizers: the PCE and potential biological toxicity of photosensitizers should be carefully considered in advance. For examples, inorganic PTAs are known to have higher PCEs in general, but the degradation *in vivo* is still unclear. Although organic PTAs have inherent excellent biodegradability, they also suffer from the limitations of photostability and thermal stability. Besides this, PTAs that strongly absorb in the NIR-II region show superior potential due to their deep tissue penetration and high maximum permissible energy for the skin. For example, semiconducting polymers improve the possibility of clinical application.^121^ (b) Configuration choice: it has been reported that NPs of around 100 nm in size have a longer circulating time *in vivo*, meaning that it is difficult for them to clear biological barriers. Moreover, rod-shaped materials have better permeability in the tumor tissue, which is beneficial for the treatment of solid tumors. Many investigations have illustrated that organic PTAs should be synthesized into nanoscale materials. (c) Targeting ligands: given the fact that targeting ligands have different compositions and aiming positions, it is necessary to reasonably design PTAs with targeting ligands. A synergistic targeting strategy is an ideal method by which to prepare specific PTAs, however, targeting ligands should not interfere with each other to identify tumor sequentially. It is worth noting that large ligands can shield small molecule ligands resulting in decreased targeting efficiency, so this should be avoided before the design of the multifunctional PTAs. (d) Methods for the modification of targeting ligands: the methods for the modification of targeting ligands also seriously influences the efficiency of dual-targeted PTAs for cancer therapy. In general, multifunctional PTAs can be simply coated onto a targeted delivery system, which can easily cause side effects because of the risk of leakage. So, it is highly desirable to connect targeting ligands to PTAs using covalent bonds. Although synthetic methods may be more difficult than physical coating, they provides a safe strategy for *in vivo* therapy. In order to achieve optimal tumor delivery, in the preparation of an ideal dual-targeted agent the above problems should be considered in advance.

In conclusion, recent advances in dual-targeted PTT for tumors are summarized in this review. DTPTAs have provided an effective and non-invasive alternative with more accurate positioning capabilities compared to traditional PTAs, which possess the extensive advantages of low side effects and high efficiency in tumor therapy. Although scientists have made great efforts in this field, there are still many challenges to overcome before realizing clinical applications. Prospectively, good biocompatible DTPTAs have low toxicity to organisms, and will be superior choices for clinical application. We believe that this report will provide valuable ideas and references for researchers to enable them to design novel DTPTAs to enhance cancer therapy.

## Conflicts of interest

The authors declare no competing financial interests.
